# Neuroinflammation and Mitochondrial Dysfunction in Parkinson’s Disease: Connecting Neuroimaging with Pathophysiology

**DOI:** 10.3390/antiox12071411

**Published:** 2023-07-12

**Authors:** Benjamin Matís Pizarro-Galleguillos, Liesa Kunert, Norbert Brüggemann, Jannik Prasuhn

**Affiliations:** 1 Facultad de Medicina, Universidad de Chile, Santiago 8380453, Chile; benjaminpizarro@ug.uchile.cl; 2 Department of Neurology, University Medical Center Schleswig-Holstein, Campus Lübeck, 23562 Lübeck, Germany; liesa.kunert@neuro.uni-luebeck.de (L.K.); jannik.prasuhn@neuro.uni-luebeck.de (J.P.); 3 Institute of Neurogenetics, University of Lübeck, 23562 Lübeck, Germany; 4 Center for Brain, Behavior, and Metabolism, University of Lübeck, 23562 Lübeck, Germany; 5 Russell H. Morgan Department of Radiology and Radiological Science, Johns Hopkins University School of Medicine, Baltimore, MD 21205, USA; 6 F.M. Kirby Research Center for Functional Brain Imaging, Kennedy Krieger Institute, Baltimore, MD 21287, USA

**Keywords:** Parkinson’s disease, neuroinflammation, mitochondrial dysfunction, magnetic resonance imaging (MRI), magnetic resonance spectroscopy imaging (MRSI), positron emission tomography (PET) imaging, TSPO, mitochondria, neuroimaging

## Abstract

There is a pressing need for disease-modifying therapies in patients suffering from neurodegenerative diseases, including Parkinson’s disease (PD). However, these disorders face unique challenges in clinical trial designs to assess the neuroprotective properties of potential drug candidates. One of these challenges relates to the often unknown individual disease mechanisms that would, however, be relevant for targeted treatment strategies. Neuroinflammation and mitochondrial dysfunction are two proposed pathophysiological hallmarks and are considered to be highly interconnected in PD. Innovative neuroimaging methods can potentially help to gain deeper insights into one’s predominant disease mechanisms, can facilitate patient stratification in clinical trials, and could potentially map treatment responses. This review aims to highlight the role of neuroinflammation and mitochondrial dysfunction in patients with PD (PwPD). We will specifically introduce different neuroimaging modalities, their respective technical hurdles and challenges, and their implementation into clinical practice. We will gather preliminary evidence for their potential use in PD research and discuss opportunities for future clinical trials.

## 1. Introduction

Parkinson’s disease (PD) is the fastest growing neurodegenerative disorder and an unprecedented challenge for the healthcare system of aging societies [1]. Besides recent advancements in developing symptomatic treatment regimes, no causative therapies are available to date [1]. PD onset and progression result from a predominantly dopaminergic (DAergic) cell loss in the *Substantia nigra* (SN) [2], although the underlying causes of this neuronal loss are not fully understood. Still, it is presumed to involve a complex interplay of genetic and environmental factors, neuroinflammation, and mitochondrial dysfunction [3]. Neuroinflammation is a complex process that involves the activation of immune cells in the brain, the production of inflammatory mediators such as cytokines and chemokines, and the activation of other cell types in response to neuronal damage or injury [4]. In animal models, research has shown striking evidence of neuroinflammation as a pathophysiological driver of neurodegeneration in PD, including the activation of microglia and resident immune cells of the brain, and the production of pro-inflammatory cytokines [4]. Several observational studies have investigated the relationship between neuroinflammation and PD [5,6], showing that PwPD had higher levels of inflammatory markers in their blood and cerebrospinal fluid (CSF) than healthy controls (HCs) [5,7,8]. Furthermore, several genetic risk markers or mutations in PD-causing genes are involved in regulating the immune system, suggesting a potential role for neuroinflammation in inherited forms of PD [9,10].

**Figure 1:**
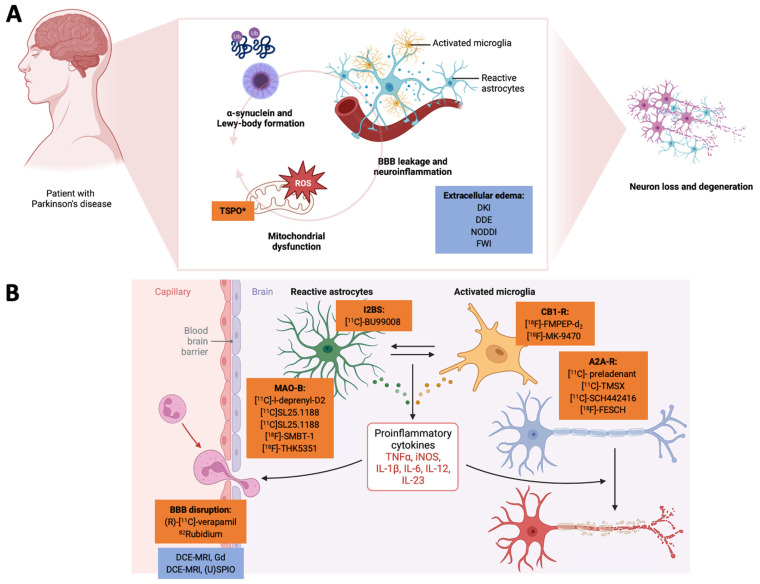
Here, we highlight the interconnected nature of neuroinflammation and mitochondrial dysfunction and how pathophysiology-orientated neuroimaging can be applied to study these disease mechanisms. In Panel (**A**), we demonstrate how a-synuclein deposition, mitochondrial dysfunction, and the tissue/cellular aspects of neuroinflammation act together in a vicious cycle to promote neurodegeneration. Radiotracers are highlighted in orange, and MRI-based neuroimaging approaches are highlighted in blue. Here, TSPO can also be considered an unspecific marker of the mitochondria. BBB leakage and the extracellular edema component of neuroinflammation are usually investigated by (advanced) diffusion-weighted MRI methods. In Panel (**B**), we illustrate how (pre-)clinical radiotracers can address the cellular and humoral neuroinflammatory responses. The closeup on the BBB also highlights novel MRI-based approaches to assess BBB integrity. *TSPO can be interpreted as a marker of neuroinflammation but also for mitochodrial membrane integrity. (U)SPIO: (ultra-)small paramagnetic iron oxide particles. A2A-R: adenosine receptor type 2A. BBB: blood–brain barrier. CB1-R: cannabinoid receptor type 1. DCE-MRI: dynamic contrast-enhanced MRI. DDE: double diffusion encoding imaging. DKI: diffusion kurtosis imaging. FWI: free-water imaging. Gd: gadolinium. I2BS: imidazoline-2 binding sites. IL: interleukin. iNOS: induced nitric oxide synthase. MAO: monoamine oxidase. NODDI: neurite orientation dispersion and density imaging. TNF: tumor necrosis factor. TSPO: mitochondrial translocator protein. Created with biorender.com.

Recent research has suggested that neuroinflammation and mitochondrial dysfunction may be interconnected disease mechanisms in PD (Figure 1). For example, pro-inflammatory cytokines can impair mitochondrial function via dysregulating oxidative phosphorylation (OXPHOS) and causing oxidative stress [3,10,11]. Mitochondrial dysfunction can also activatate immune cells and further promote neuroinflammation [3,10,11]. The temporal dynamics are unclear, but there appears to be a self-promoting interaction between both [3,10,11]. Interestingly, many genetic risk factors or disease-causing genes in PwPD are not only involved in the regulation of the immune system but also mitochondrial homeostasis, suggesting a potential link between these processes in inherited forms of PD. Prominent examples are the genes *Parkin* and *PINK1* that have previously been implicated in mitochondrial homeostasis via regulating the clearance of dysfunctional mitochondria, a process called mitophagy [12]. Mechanistic studies in animal models and humans have highlighted the release of damage-associated molecular patterns that can activate innate immunity, suggesting that mitophagy may mitigate neuroinflammation [13]. This is further supported by elevated pro-inflammatory blood biomarkers in individuals with mono- and biallelic *Parkin* mutations [14]. Here, activating *the cyclic GMP-AMP synthase/stimulator of interferon genes* pathway mitigated the activation of the innate immune system, which was demonstrated by elevated interleukin-6 levels [13,14]. However, this is only one example of the highly interwoven nature of neuroinflammation and mitochondrial dysfunction in PwPD. This molecular complexity opens a unique window of opportunity for pathophysiology-orientated neuroimaging approaches in studying PwPD (Figure 1).

Neuroimaging-based methods are widely used in research and clinical settings to investigate neuroinflammation in the diseased brain, providing valuable information on the location, extent, and severity of neuroinflammatory processes. Neuroimaging provides the ability to visualize in vivo not only neuroanatomical and functional signatures but also pathophysiology-related pathways such as neuroinflammation and mitochondrial dysfunction, and potentially allow the development of disease-specific biomarkers [15].

In this narrative review, we will focus on positron emission tomography (PET) and advanced magnetic resonance (spectroscopy) imaging (MRI/MRSI) methods that can be used to study neuroinflammation and mitochondrial dysfunction. In this way, the development of reliable neuroimaging biomarkers not only gives insights into highly relevant disease mechanisms but could potentially serve as clinical outcome variables in clinical trials, facilitate diagnostic and prognostic tests, evaluate early pathophysiology-targeted interventions, estimate disease stages and the rate of disease progression, inform treatment decisions, and could potentially map treatment responses [16,17,18,19]. Moreover, neuroimaging-based approaches can disentangle the intra- and inter-disease heterogeneity of neurodegenerative disorders, i.e., PD and atypical forms of parkinsonism [16,19,20].

Although MRI is mainly applied to gain insights into the brain’s structure, novel methods can also assess molecular phenomena, such as advanced diffusion metrics to investigate blood–brain barrier (BBB) integrity, extracellular edema, alterations of cerebral bioenergetics, and the presence of oxidative stress. While neither of these neuroimaging methods provide information at the cellular level, PET imaging allows the characterization and visualization of specific molecules, MRI measures biophysical properties of brain parenchyma, and MRSI can detect molecules and metabolic pathways at the tissue level, all closely related to neuroinflammation and mitochondrial dysfunction [21].

## 2. Main Body

In this review, we mainly describe imaging techniques to track the humoral and cellular components of neuroinflammation (e.g., by mapping microglial activation/migration), BBB disruption, and methods to track the edema component of neuroinflammation. In the part on mitochondrial dysfunction, we will mainly focus on methods to map surrogate markers of OXPHOS and oxidative stress. In the following, only PET- and MRI-based approaches will be highlighted.

### 2.1. Tracking the Humoral and Cellular Components of Neuroinflammation through Positron Emission Tomography

PET is an imaging technique that uses the radioactive decay of specifically designed radiotracers. In PET imaging, the annihilation of two photons that are produced back-to-back after positron emission from the radiotracer is measured by a technique called coincidence detection. After amplifying the signal, reconstruction algorithms are used to generate the image [22]. One of the most commonly used diagnostic radiotracers in patients with neurodegenerative disorders (PwND) is [^18^F]-fluorodeoxyglucose, which serves as a surrogate marker of glucose metabolism [23]. PET imaging has been extensively used in several movement disorders, including PD [24] and atypical parkinsonism [25,26]. Within the scope of neuroinflammation, PET imaging has been used for detecting microglia and astrocyte activation [27], the expression levels of adenosine receptors [28], as well as other neuroinflammatory molecules [29], but also oxidative stress and mitochondrial dysfunction [30]. Even though the current understanding of neuroinflammation underlines its complexity, PET imaging can help to disentangle specific components of the neuroimmunological response [29]. One of the most commonly applied PET radiotracers in studying neuroinflammation is targeting the mitochondrial translocator protein (TSPO). However, other potential molecular targets for PET radiotracer development have been identified, including monoamine oxidase type B (MAO-B), cannabinoid receptor type 1/2 (CB1-/CB2-R), and phosphodiesterase type 4 [31]. In addition, the cellular components of neuroinflammation have been investigated using radiotracers targeted against cyclooxygenase (COX, as a marker of resting microglia), thrombospondin, COX type 2, and P2X purinoreceptor 7 (P2XR7, as a marker of activated microglia). In addition, TSPO and MAO-B have also been considered proxies for astrocyte activation [29]. The development of new molecule-specific radiotracers is a highly dynamic field of research. For example, new radiotracers have been developed targeting glycogen synthase kinase 3 (GSK-3), phospholipase A2/arachidonic acid pathway, sphingosine-1-phosphate receptor-1, the chemokine receptor CX3CR1, additional purinoreceptors, receptors for advanced glycation end products, proto-oncogene tyrosine-protein kinase MER (MERTK), and triggering receptor expressed on myeloid cells-1 (TREM-1) [32,33]. Many of these molecular targets recapitulate unique features of neuroinflammation. The following paragraphs will focus on the more widely applied radiotracers in neurodegenerative disorders research (Supplementary Tables S1 and S2).

#### 2.1.1. The Mitochondrial Translocator protein in the Context of Mapping Neuroinflammation

TSPO is an 18 kDa protein mainly localized in the outer mitochondrial membrane [34]. The overall concentration of TSPO in mammalian brains is low, but under neuroinflammatory conditions, i.e., following glial activation, the expression of TSPO is greatly enhanced [35]. TSPO is thought to be involved in many cellular pathways, including neurosteroid synthesis, apoptosis signaling, mitochondrial bioenergetics, and ROS processing [36]. However, the precise role of TSPO is still under investigation [31]. Even though TSPO has been recognized as a marker of astrocyte activation, it is also expressed in microglia, vascular endothelial cells, neurons, and immune cells [31]. Several radiotracers have been developed for detecting TSPO expression. These TSPO-targeted radiotracers can be classified into first- and second-generation radiotracers according to their improved pharmacodynamics and overall sensitivity to detect neuroinflammatory changes in brain parenchyma [32].

[^11^C]-PK11195 is a first-generation TSPO-radiotracer which has been most widely used in studies investigating neuroinflammation in PwND [37]. Despite the limitations of [^11^C]-PK11195, including poor BBB permeability, non-specific binding, and a relatively low signal-to-noise ratio, it has given valuable insights into PD [32]. In a recent meta-analysis, nine studies using [^11^C]-PK11195 in PwPD have been identified [35]. Here, PwPD had significantly higher TSPO levels than HCs in several brain regions, including the midbrain, basal ganglia, cerebellum, thalamus, hippocampus, and cortical areas. Interestingly, the most significant effect was observed in the temporal lobe of PwPD [35]. However, the authors stressed the heterogeneity observed in several neuroanatomical regions [35].

In the same meta-analysis, the authors also included five studies employing second-generation TSPO radiotracers ([^18^F]-FEPPA, [^11^C]-DPA713, [^11^C]-PBR28, [^11^C]-DPA714) [35].

Here, a pooled analysis revealed significantly higher TSPO levels in the midbrain of PwPD compared to HCs. In contrast to the previously reported results on first-generation TSPO radiotracers, other neuroanatomical regions no longer exhibited relevant group differences [35].

The consistent TSPO overexpression in the midbrain of PwPD is well in-line with our understanding of a predominant DAergic neuronal loss in the SN. Here, TSPO-targeted PET imaging already demonstrated the potential involvement of neuroinflammation in PD pathophysiology [35]; however, TSPO-targeted PET imaging faces several challenges. Even though often considered a proxy of astrocyte activation, TSPO-targeted radiotracers lack cellular specificity. In addition, microglial activation can be beneficial or detrimental in the fine-tuned regulation of neuroinflammation, mainly due to the involved microglia subtype [32]. Besides the improvements of first-generation TSPO radiotracers’ drawbacks, the widespread application of these tracers has also been limited by several factors, e.g., the presence of genetically-encoded TSPO polymorphism that can affect radiotracer-binding [38]. Interestingly, TSPO tracers have shown the potential as a treatment response marker [39].

#### 2.1.2. The Involvement of Astrocytes in Neuroinflammation: The Potential Role of Monoaminoxidase B and Imidazoline-2 Binding Sites in Radiotracer Development

In general, activated astrocytes and microglia have been observed in many PwND [40]. The involvement of astrocytes in PD is undisputed [4]. Post-mortem studies have shown cytoplasmatic alpha-synuclein (aSyn) inclusions also aggregate in astrocytes [41]. Astrocytes either react or contribute to neurodegenerative processes by changing morphology and secreting proinflammatory cytokines, similar to microglia [40,42].

Astrocytes express high levels of MAO-B. Thus, this molecule has been proposed as a neuroimaging biomarker of astrogliosis. Post-mortem studies have further demonstrated that MAO-B levels are elevated in the frontal cortex of post-mortem PwPD but not in the SN [43]. MAO-B-targeted radiotracers have already been used in PwPD and other ND [44], with [^18^F]-THK5351 being the first. Originally, [^18^F]-THK5351 was designed to detect tau aggregates. However, [^18^F]-THK5351 showed higher binding affinities to MAO-B [40]. In a comparative study, [^18^F]-THK5351 was applied to differentiate PwPD from patients with progressive supranuclear palsy (PwPSP) and the cerebellar type of multiple systems atrophy (PwMSAc). Here, diencephalic and midbrain [^18^F]-THK5351 uptake differentiated PwPD from PwPSP. In contrast, pontine and cerebellar uptake of [^18^F]-THK5351 appeared specific for PwMSAc [45]. Even though there is strong evidence for the interconnected nature of MAO-B activity and mitochondrial dysfunction, there is a significant lack of [^18^F]-THK5351 studies in PwPD [46]. Considering that the role of MAO-B inhibition is part of the therapeutic arsenal in PwPD, this observation is even more surprising [46]. This also extends to more advanced MAO-B-targeted radiotracers ([^11^C]-l-deprenyl-D2, [^11^C]SL25.1188, and [^18^F]-SMBT-1), where no studies in PwPD were found (Supplementary Tables S1 and S2).

The imidazoline-2 binding sites (I2BS) are located on the cell membrane of astrocytes and are expressed in the cortex, hippocampus, basal ganglia, and brainstem, making them suitable targets for studying PwPD [41]. One study in 22 PwPD and 14 HCs used [^11^C]-BU99008 as a highly selective I2BS radiotracer [41]. Here, early-stage PwPD exhibited increased [^11^C]-BU99008 uptake in the frontal, temporal, parietal, and occipital cortex. However, a higher [^11^C]-BU99008 uptake was seen in the brainstem of PwPD compared to HCs [41]. On the other hand, late-stage PwPD showed a comparable [^11^C]-BU99008 uptake pattern, but also in the insula, the basal ganglia, the thalamus, and the brainstem [41]. The authors observed that [^11^C]-BU99008 radiotracer uptake correlated with motor and non-motor symptom severity [41]. These results encourage the use of new radiotracers tracking astrocytosis. However, more research is needed to elucidate further and replicate the above-stated findings longitudinally in larger cohorts [27].

#### 2.1.3. The Endocannabinoid System in Neuroinflammation: Opportunities for Neuroimaging Theranostics

Recent studies have highlighted the endocannabinoid system as a relevant regulator in the CNS, including processes such as neuroinflammation and neurogenesis [47]. Different cannabinoid receptors are known. However, the cannabinoid receptor type 2 (CB2-R) has been previously implicated in neuroinflammation [33]. Rodent microglia express CB2-R under normal conditions. The initiation of neuroinflammatory treatments produces a marked CB2-R upregulation in rodents [33]. Studies in PwPD also reveal increased CB2-R levels in SN microglia, corroborating these preclinical findings [48]. Despite the potential role of CB2-R in PwPD, no studies have been found exploring CB2-R-targeted radiotracers in PwPD. This exciting approach may also extend to the cannabinoid receptor type 1 (CB1-R) being highly expressed in the basal ganglia, making it interesting to study the pathophysiology of PD [49]. The radiotracer [^18^F]-FMPEP-d_2_ has been applied to map CB1-R distribution in vivo [49]. This approach has also been evaluated by the use of [^18^F]-MK-9470 in a 6-hydroxydopamine (6-OHDA) animal model [50] and also in PwPD using the same radiotracer [51]. In summary, these studies have shown decreased subcortical CB1-R availability, but the biological underpinnings of these findings require further research. Current interpretations include the role of CB1-R in coping with oxidative stress and neuroinflammatory excitotoxicity. Thus, further research is necessary to evaluate the role of CB1-R as a specific target to reduce neuroinflammation [50] (Figure 1).

#### 2.1.4. Propagating Neuroinflammation: The Role of Adenosine Receptors in Precision Imaging

Adenosine receptors are purinergic G-protein coupled receptors broadly expressed in the peripheral and central nervous system (CNS) [52]. In the case of neuroinflammatory processes, neurons and glial cells can release adenosine, which can confer local effects through purinergic receptors leading to the secretion of pro-inflammatory cytokines, activation, and migration of microglia, and alterations in astrocyte function [53]. A2A receptors are highly expressed on neuronal surfaces in the striatum [54]. Several studies have used PET radiotracers targeting these receptors in animal models [55] and humans [56,57,58,59,60]. Even though studies have pointed toward the involvement of adenosine signaling in neuroinflammation, conclusive evidence in humans is scarce as the main use of A2A receptor-targeted radiotracers has been within the scope of their colocalization to D2 receptors [52].

#### 2.1.5. Novel Radiotracers on the Brink of Mapping Neuroinflammation

Despite the (pre-)clinical availability of highly specific radiotracers, studies in PwPD on humoral and cellular aspects of neuroinflammation are still generally rare. For example, the role of GSK-3 beta has been implicated in the pathophysiology of PwPD and is considered a potential treatment target [61]. Albeit, GSK-3-specific radiotracers have not yet been evaluated in PwPD. Another example includes COX-2-targeted radiotracers, assessed in PwND but not PwPD [62]. Even if arachidonic acid has been considered a central part of the neuroinflammatory cascade in PwPD, including the synthesis of prostaglandins and leukotrienes, and 1-[^11^C]-arachidonic acid can be considered a promising radiotracer [63], no studies in PwPD have been performed so far. Sphingosine-1-phosphate receptor 1 is a G protein-coupled receptor that is highly overexpressed during the neuroinflammatory response, and [^11^C]-TZ3321 is a specific tracer for this molecule. However, only one short report in nonhuman primates as a PD animal model was found [64]. In addition, no studies using TMERK- or TREM1-specific radiotracers in PD research were found. This opens up exciting opportunities for evaluating these new or unused radiotracers, which could complement our current understanding of the complex role of neuroinflammation in PwPD.

### 2.2. Mapping Blood–Brain Barrier Disruption by Multi-Method Neuroimaging

The BBB separates the neuronal brain parenchyma of the CNS from the blood. The term BBB describes a series of cellular components and subsequent functionalities of the vasculature reaching the CNS. The BBB tightly regulates the transport of ions, molecules, and cells between the blood compartment and the CNS, including many components relevant to neuroinflammation [65]. Therefore, BBB disruption has been a research focus in neurovascular diseases and PwND as part of the neuroinflammatory pathophysiology [66].

Studies in rodent models have highlighted the involvement of the BBB as part of neuroinflammatory processes with an increased BBB permeability [67]. Some PET radiotracers, such as (R)-[^11^C]-verapamil, have been used as a proxy measure of the P-glycoprotein (P-gp) function [67]. P-gp is an ATP-dependent efflux pump with broad substrate specificity and is necessary to remove foreign substrates from BBB cells [67]. However, decreased BBB P-gp function in early-stage PwPD could not be confirmed [68]. Recent studies have used ^82^Rubidium and PET to quantify BBB influx in vivo. Consistently, no significant changes in BBB permeability in PD patients were found with this approach [67]. However, BBB disruption may still play an essential role in PwPD, assuming that PET-based neuroimaging methods within this scope face several limitations.

Therefore, several MRI-based methods have been developed to study BBB disruption via completely different image contrast-generating approaches. MRI provides a noninvasive technique to assess BBB disruption, with other techniques available, including gadolinium (Gd)-based dynamic contrast-enhanced MRI (DCE-MRI), (ultrasmall) superparamagnetic particles of iron oxide (USPIO/SPIO) MRI, and arterial spin labeling (ASL) [69]. However, ASL has been mainly used for brain perfusion studies, and no studies in PwPD were found specifically assessing ASL for probing BBB disruption. In the following, we will focus on DCE-MRI and (U)SPIO-MRI.

Quantitative information about the functional integrity of the BBB can be gained by performing DCE-MRI [70]. DCE-MRI visualizes tissue’s dynamic enhancement in response to administering a contrast agent, such as GDd, into the vascular system. What distinguishes DCE-MRI from other neurovascular imaging modalities, such as blood-oxygen level-dependent imaging (BOLD) or ASL, is its unique ability to quantitatively measure other microvascular parameters, such as vessel permeability and fluid volume fractions. The former is particularly relevant to the neuroinflammatory process [71]. Gd is assumed to diffuse passively within the interstitium along a concentration gradient as a small molecular weight contrast agent. This is also the case following neuroinflammation-related BBB disruptions [72]. It has already been known for a long time that local Gd enhancement correlates with histological markers of BBB breakdown [73]. In this line of argumentation, Gd enhancement parallels BBB leakage [74]. However, this technique can only be considered a reductionist approach to the complexity of neuroinflammation [75] (Figure 1).

DCE-MRI may overcome some of these oversimplification-related shortcomings of standard Gd-enhanced MRI. Two animal studies addressed the role of BBB leakage in PD models using DCE-MRI [76,77]. Here, the authors demonstrated the interconnected nature of iron deposition, BBB disruption, and neuroinflammation in a 6-OHDA rat model [76]. DCE-MRI showed increased SN-localized BBB disruption in the first week after the 6-OHDA injection, with the restoration of BBB disruption in the 6-OHDA animal model but not in control animals [76]. Furthermore, immunohistochemical analyses demonstrated IgG extravasation, with no immunoreactivity observed after four weeks in the PD animal group [76].

Moreover, T2*-weighted images demonstrated increased SN iron deposition, further confirmed by an ex vivo MRI analysis and immunohistochemistry [76]. Histopathological examinations additionally have proven the extravasation of proinflammatory CD68^+^-cells, which also colocalized with iron deposition [76]. The second study combined DCE-MRI with USPIO-MRI and will be discussed more in-depth in the following paragraphs.

In humans, one study using DCE-MRI compared quantitative maps of contrast agent transfer coefficient across the BBB (in the following named K^trans^) and plasma volume using a voxel-wise and also a region-of-interest (ROI)-based approach [66]. Here, 49 PwPD, 15 disease-controls suffering from small-vessel disease, and 31 HCs were enrolled [66]. Voxel-wise analyses showed higher K^trans^ differences in the posterior white matter (WM) regions of PD compared to HCs but not in the disease-control group [66]. Furthermore, ROI-based analyses confirmed significantly higher K^trans^ in the SN of PwPD versus HCs in normal-appearing WM, WM lesions, and the posterior cortex [66]. However, the group also assessed the WM lesions burden in PD, showing a similar WM lesion burden with the control positive group undermining this approach [66]. In this way, even if BBB disruption was observed, these findings’ pathophysiology underpinnings will need further investigation. Another study used DCE-MRI to study the meningeal lymphatic flow (often referred to as the glymphatic system) in PwPD, atypical parkinsonism, and HCs [78]. In this work, PwPD showed a reduced meningeal lymphatic flow along the sigmoid and superior sagittal sinuses [78]. In this study, the authors also investigated whether the disease stage affected the glymphatic system. PwPD were divided into two subgroups (Hoehn and Yahr Stage ≤ 2.5 and > 2.5) [78]. This subgroup analysis suggested that advanced PwPD demonstrates a more severe impairment of the glymphatic flow [78]. Interestingly, the authors also used these measures as a neuroimaging biomarker for the differential diagnosis of PD in contrast to patients with atypical parkinsonism, showing a sensitivity of 93% and a specificity of 97% [78]. Nevertheless, the conclusion of the previous work should be interpreted with caution, as this study provides only indirect evidence that meningeal lymphatic dysfunction in PwPD is associated with neuroinflammation.

(U)SPIO-MRI uses iron oxide nanoparticles as a contrast agent for contrast-enhanced MRI [79]. The classification of these nanoparticles is mainly based on their size. Regular SPIO have an average diameter of 50–150 nm and USPIO of <50 nm [74,80,81]. In recent years, SPIO and USPIO have shown relevant applications in diagnosing various conditions, such as neurovascular diseases and inflammatory changes from malignant tumors [82]. Compared to Gd as a contrast agent, (U)SPIOs provide unique benefits concerning biocompatibility [83]. (U)SPIOs tend to be recognized and phagocytosed by the reticuloendothelial system. Therefore, (U)SPIOs can be used to track the presence and migration of phagocytes in the CNS as a marker of BBB disruption [82]. This has been shown for vascular leakage in tumors [82]. Later evidence suggests that macrophages can also cross the intact BBB, making them more sensitive to macrophage activation and not only rely on BBB disruption [77]. The former also has been confirmed by the colocalization of TSPO radiotracers with USPIO [77]. Another biological interpretation is that DCE-MRI and (U)SPIO-MRI can track the inflammatory response in different temporal stages, where monocyte infiltration into the CNS is followed by BBB disruption [77].

In a PD rat model, the authors investigated whether the presence of macrophages in the CNS was mainly determined by resident microglia or secondary to CNS invasion from circulating blood cells [77]. They further investigated whether an antioxidative diet influences the individual distribution of these immune cells using SPIO, T2*-weighted MRI, and also DCE-MRI [77]. Here, they used SPIO-labeled peripheral immune cells to demonstrate the migration of these cells into the CNS. This migration appeared without DCE-MRI-detected BBB disruption [77]. The authors interpreted these findings as macrophage migration without neurovascular permeability changes as a driver of neuroinflammation in their PD rat model [77].

Interestingly, the authors also observed a higher rate of CNS invasion of SPIO-labeled macrophages following an antioxidative diet [77]. The former could be an interesting hypothesis in investigating the role of mitochondrial dysfunction in neuroinflammation. Previous studies indicated that microglia levels correlated with dopamine regeneration in mice treated with antioxidants [84].

However, (U)SPIO-MRI cannot distinguish macrophages with pro-inflammatory from anti-inflammatory profiles. To our knowledge, no studies have been performed on PwPD using (U)SPIO-MRI. No matter the scarcity of (U)SPIO-MRI studies in PD animal models or PwPD, the characteristics of the above-exposed techniques highlight the vast potential of these methods to track neuroinflammation-related phenomena such as BBB disruption and macrophage migration (Supplementary Tables S1 and S3).

### 2.3. The Role of MRI-Based Approaches in Mapping the Edema Component of Neuroinflammation

The activation of the vascular endothelium during neuroinflammation leads to increased BBB permeability, e.g., by the downregulation of tight junctions [74]. The increase in BBB permeability consequently leads to an increased passage of plasma proteins to the extracellular compartment of the CNS, defined as vasogenic edema. In contrast, the cellular water intake, especially from astrocytes, has been termed cellular edema [85].

Mapping edema is one key strength of MRI-based neuroimaging approaches. However, even if T2-weighted MRI hyperintensities could be interpreted as the presence of vasogenic edema, it still lacks quantitative parameters and specificity, so more precise methods should be considered [86]. Diffusion-weighted MRI (and diffusion tensor imaging, DTI) can provide quantitative parameters related to free-water movement and restriction in brain tissue. Despite the former, traditional DTI measurements are considered unspecific for neuroinflammation-related changes [21,87]. Continuous methodological advancements have led to advanced diffusion metrics with higher biological interpretability. In the following, we will review state-of-the-art approaches, such as diffusion kurtosis imaging (DKI), double diffusion encoding (DDE), neurite orientation dispersion and density imaging (NODDI), and free-water imaging (FWI) as suitable surrogate markers of edema related to neuroinflammation [17] (Figure 1).

#### 2.3.1. Diffusion Kurtosis Imaging

DKI signals quantify the degree of non-Gaussian diffusion associated with complex brain microstructures and have been proposed as a mathematical extension of DTI. In this way, the technique allows measuring the degree of diffusion restriction or brain tissue complexity [88]. DKI has been proposed as a sensitive marker of neuroinflammation due to the increased complexity of the extracellular space during the neuroinflammatory process [21]. Two original studies using DKI in PD mice models were identified [89,90]. In an aSyn mouse model, the authors demonstrated how DKI metrics were increased in the thalamus and sensorimotor cortex compared to wild-type mice [90].

Additionally, DKI metrics positively correlated with aSyn levels in the thalamus without such correlations seen in SN [90]. However, no direct histology-based markers of neuroinflammation were assessed [90]. In the other study, the authors used DKI to study microstructural changes in the methamphetamine-induced mouse model of PD [89], demonstrating a decrease in DKI metrics in the SN, striatum, and sensorimotor cortex as a potential marker for a loss of DAergic neurons [89]. Again, no direct measurements of neuroinflammation were investigated [89]. Although DKI has shown an association with neuroinflammatory markers in other diseases, specific preclinical studies on PD models are still needed to confirm DKI as a reliable biomarker of neuroinflammation. In contrast, DKI metrics have been associated with microgliosis during the acute inflammatory phase in other neurological diseases [91], and also in animal models of astrogliosis [92].

DKI has been repeatedly applied in PwPD. The most recent and comprehensive systematic review and meta-analysis of DKI imaging in PD [93] included 14 studies with 535 PwPD and 486 HCs. Besides some methodological heterogeneity, extensive alterations, e.g., of subcortical DKI metrics, have been observed [93]. All studies discussed these changes within the scope of microstructural changes to neuronal cell loss and also related these findings with the overall disease course and symptom severity [93]. None of the above-mentioned studies investigated DKI metrics within the scope of neuroinflammation [93].

#### 2.3.2. Double Diffusion Encoding

DDE can provide additional information not contained in the standard diffusion models of DTI and DKI: *microscopic anisotropy* and the *variance of the isotropic diffusivities* among individual microenvironments [94]. In a comparative study of DKI and DDE, the reduction of DKI metrics reported in most PwPD studies was mainly driven by a decrease in microscopic anisotropy [95]. These findings also correlated with motor symptom severity. However, a direct connection between this marker and neuroinflammation was lacking, even though it recapitulates edema and inflammation-related changes in the tissue microstructure. However, the ongoing application of DDE may substantially foster the interpretability of DTI-derived findings in previous studies.

#### 2.3.3. Neurite Orientation Dispersion and Density Imaging

NODDI is an advanced MRI diffusion technique that probes the microstructure of neurites [96]. It uses a three-compartment model, including intracellular volume, extracellular volume, and CSF [96]. This technique quantifies unique diffusion metrics, such as isotropic free-water, intracellular volume fraction, neurite density, dispersion of neurites, and the orientation dispersion index (ODI) [97]. NODDI has been proposed to overcome DTI- and DKI-related limitations, such as Gaussian water movement assumptions and the nonspecificity of DTI scalars, such as the fractional anisotropy (FA) [97]. Some research groups have tried to demonstrate the biological interpretability of NODDI-derived parameters with neuroinflammation, with promising results [98,99].

As described previously, microglial activation is a reliable biomarker of neuroinflammation. Research in C57BL/6J male mice has shown that ODI positively correlated with the microglial density in brain parenchyma following colony-stimulating factor 1 receptor inhibitor treatment [99]. This has also been recapitulated in patients with MS where a higher ODI was observed in gadolinium-enhancing brain lesions [100]. Several studies have already applied NODDI to investigate PwPD [96,101,102,103,104,105,106]. Here, NODDI-derived parameters have demonstrated potential use in the differential diagnosis of parkinsonian disorders, the overall assessment of the disease course, the predominant symptoms, and (non-)motor symptom severity [96,101,102,103,104,105,106]. Again, methodological inconsistencies led to heterogeneity in the observed results. Even if these results are promising and could be useful as a PD biomarker, further research on direct measures of neuroinflammation and its correlation with NODDI-derived metrics are needed [95].

#### 2.3.4. Free-Water Imaging

FWI is a diffusion technique that uses a two-compartment model capable of quantifying extracellular free-water and eliminating signals from CSF. This technique can separate the diffusion properties of the brain tissue from surrounding free-water while mapping the free-water volume as estimated from a regularized bi-tensor model [107].

Transgenic mice overexpressing interferon-y exhibit increasing levels of free-water in white matter structures, midline cortical areas, and the medial thalamic areas, suggesting a correlation between this biomarker and neuroinflammation [108].

Several studies have illustrated the applicability of FWI in PwPD, with most studies showing a consistent increase in the amount of free-water in the SN of PwPD [96,102,109,110,111,112,113,114,115,116]. Most studies focused on the posterior portion of the SN (pSN), containing the nigrosome-1. As such, the precise distribution of the SN free-water signal is still under research [113]. FWI has also demonstrated alterations in other brain regions, such as the caudate and putamen. However, the results for these regions are heterogeneous [117]. FWI has been used as a progression marker within a randomized controlled trial investigating the potential neuroprotective properties of Rasagiline in early-stage PwPD [118]. Even though the authors did not find any treatment-related group differences, the free-water signal increased in the pSN after one year and correlated with the overall clinical progression of motor symptoms [118]. However, these findings contradict previous results from observational studies [111]. A recent study compared SN iron content, free-water, and diffusion metrics in the SN of moderate-stage PwPD using T2* relaxometry, single, and bi-tensor models of diffusion-weighted MRI [119]. Here, the free-water signal was increased in the pSN of PwPD compared to HCs, while R2*-values were increased in the anterior portion of the SN [120]. The interpretation of R2*-values as a surrogate marker of iron deposition (as well as other measures, e.g., quantitative susceptibility-weighted imaging-derived values) point towards a potential role in neuroinflammation and mitochondrial dysfunction in PwPD [121,122]. Combining more traditional diffusion metrics with FWI may help disentangle the temporal course of neurodegeneration. In a recent study, the authors aimed to discriminate between neuroinflammation and neurodegeneration in PwPD [102]. Here, the free-water signal was compared to FAt (corrected fractional anisotropy), MDt (corrected mean diffusivity), and RDt (corrected radial diffusivity). FAt, MDt, and RDt were surrogate markers of neurodegeneration [102]. PwPD showed a lower index of neurodegeneration in anterior WM while showing an increase of the free-water signal in posterior WM and gray matter (GM), corresponding to Braak Stage 4 [102]. These results encourage the potential role of the free-water signal as an in vivo biomarker of disease progression and illustrate that neuroinflammation may precede neurodegeneration [102]. This has also been stressed by a study combining FWI and [^11^C]-dihydro tetrabenazine (DTBZ) PET [117]. Here, the free-water signal in the pSN has shown an inverse relationship with DTBZ radiotracer binding in the putamen and caudate [117]. The pSN-free-water signal has also been a predictor of motor symptom severity and disease stage [117]. These results are similar to previous findings [110]. Despite these promising results, most previous studies did not perform other measurements to confirm the association between FWI and neuroinflammation (Supplementary Tables S1 and S3).

### 2.4. Imaging Cerebral Bioenergetics and Oxidative Stress as Surrogate Markers of Mitochondrial Dysfunction

Oxidative phosphorylation (OXPHOS) can be considered the end route of many cellular metabolic processes (including upstream metabolic pathways, e.g., the tricarboxylic acid cycle and the non-anaerobic adenosine triphosphate, ATP, generation) [123]; however, we will restrict ourselves to measurable disruptions of OXPHOS [124]. The interested reader may also refer to previous review articles highlighting the role of pathophysiology-orientated neuroimaging in PwND [121,125].

So far, PET radiotracer development has focused on mapping nicotinamide adenine dinucleotide (NADH) ubiquinone oxidoreductase (mitochondrial complex I) distribution and activity. Here, the radiotracer [^18^F|-BCPP-EF already entered clinical evaluation, also in PwPD. Decreased [^18^F|-BCPP-EF binding has been observed in many neuroanatomical regions implicated in the pathophysiology of PD, however, without reaching statistical significance [126]. In addition, the longitudinal assessment of the same patient cohort only showed a trend without reaching significance levels [126].

The most eminent imaging modality in mapping OXPHOS-disturbances is ^31^phosphorus magnetic resonance spectroscopy imaging (^31^P-MRSI) [125]. This method allows for measuring high-energy phosphorus-containing metabolites, such as ATP or phosphocreatine (PCr) [125]. Usually, ATP and PCr are considered together to evaluate the bioenergetic state of brain parenchyma as they form a highly dynamic equilibrium in vivo [125]. Many metabolic pathways contribute to the overall generation of ATP, but the majority arise from OXPHOS, making it a suitable surrogate marker of mitochondrial dysfunction in PwPD [125]. Previous research has shown how that ^31^P-MRSI could detect bioenergetic deficits in monogenic and idiopathic PD, in contrast to atypical forms of parkinsonism [20,127]. However, ^31^P-MRSI studies often lack standardization, and multisite studies are missing [125]. ^31^P-MRSI can be combined with magnetization transfer contrast sequence designs to gain dynamic insights into ATP synthesis via physiological modeling [127,128]. One limitation of ^31^P-MRSI is the need for high (≥3 T) static magnetic field strengths and specific head coils/amplifiers, often with different architectures, which substantially limits the spatial resolution and comparability of other studies. Previously, surface head coils have been applied in PwPD, which offer only a broad signal, e.g., from the occipital lobe [127]. Interestingly, ^31^P-MRSI has been demonstrated as a dynamic biomarker of cerebral bioenergetics following experimental challenges (i.e., checkerboard paradigms to alter the bioenergetics of the occipital lobe) of treatments with mitochondrial enhancers [127,129]. In addition, the ^31^P-MRSI-based identification of the NADH redox state has also been demonstrated in humans as a surrogate marker of mitochondrial complex I activity [130].

In addition, several neuroimaging methods have been developed to map oxygen metabolism as a surrogate marker of cytochrome c oxidase (mitochondrial complex IV) activity. For decades [^15^O]-PET and ^17^O-MRSI have been used to assess the cerebral metabolic rate of oxygen consumption [125]. However, these methods have unique technical and logistical hurdles (e.g., factoring of ^17^O-containing inhalation gases), substantially hindering their use in clinical research [125]. Due to these challenges, advanced MRI methods have been developed to gain further insights into cerebral oxygen consumption using physiological models: BOLD-dependent functional MRI using respiratory challenges, T2-relaxation-under-tagging, susceptibility-based oximetry, and quantitative BOLD imaging to determine the cerebral venous blood volume and deoxyhemoglobin concentrations from T2 or T2* [125]. Our previous research article [129] discusses these methods and their respective limitations.

Proton magnetic resonance spectroscopy imaging (^1^H-MRSI) is the most widely applied in vivo spectroscopy method to study PwPD. Most ^1^H-MRSI studies in PwPD focused on the role of lactate as the end product of anaerobic metabolism to demonstrate potential mitochondrial dysfunction [131]. However, recent advancements in chemical exchange saturation transfer (CEST) imaging provide the same information with substantially higher spatial resolution [123]. However, this method has not yet been applied in PwPD.

The PET radiotracer [^62^Cu]-ATSM has been evaluated as a proxy of oxidative stress in vivo [30]. In PwPD, striatal [^62^Cu]-ATSM radiotracer uptake correlated with the disease severity [132]. Advancements in MRI sequence design led to the development of spectral-edited ^1^H-MRSI. Here, glutathione and ascorbate can be measured in vivo and have been applied in rodent models and PwPD [133,134,135]. Both metabolites play a substantial role in coping with oxidative stress [135,136].

QUEnch-assiSTed MRI (QUEST-MRI) has been proposed as an MRI-based method to map oxidative stress in vivo [137]. QUEST-MRI measures a reduction in 1/T1 (i.e., R1) to map the altered production of endogenous, paramagnetic reactive oxygen species (ROS) following antioxidative treatments [137]. However, this method has not yet been applied to PwPD.

Even though many exciting neuroimaging methods are currently under investigation, no conclusive recommendation for neuroimaging-based measures of mitochondrial dysfunction can be made. However, this is highly desirable for the development of pathophysiology-targeted treatments where these approaches could be incorporated in innovative trial designs [138,139,140]

## 3. Conclusions

In this review, we discussed neuroimaging methods that are suitable for studying the interconnected nature of neuroinflammation and mitochondrial dysfunction in the pathophysiology of PD. This shared road to neurodegeneration has been highlighted by in vitro and in vivo studies, further supported by epidemiological evidence to support their role in PD onset and progression. We summarized already established but also innovative neuroimaging methods to map key features of neuroinflammation and mitochondrial dysfunction in PwPD but also stated their respective shortcomings. While neuroimaging techniques provide exciting avenues for the early detection of PD, it is crucial to note that most of these modalities still remain in experimental stages. Large-scale clinical trials validating their efficacy in the early detection of PD are largely absent. This lack of validation introduces an element of uncertainty when interpreting the potential role of these techniques in routine clinical practice. Nonetheless, the preliminary findings offer valuable insights into the pathophysiological underpinnings of PD and suggest promising potential that warrants further exploration. To our surprise, multi-methodological studies (e.g., by combining PET imaging for neuroinflammation and MRI-based approaches for mitochondrial dysfunction) are widely lacking. However, these types of multimodal approaches would not only help to improve our current understanding of PD pathophysiology but could also yield relevant information for, e.g., patient stratification within the scope of pathophysiology-targeted clinical trials. While neuroimaging techniques provide exciting avenues for the early detection of PD, it is crucial to note that most of these modalities remain in experimental stages. Large-scale clinical trials validating their efficacy in the early detection of PD are largely absent. This lack of validation introduces an element of uncertainty when interpreting the potential role of these techniques in routine clinical practice. Nonetheless, the preliminary findings offer valuable insights into the pathophysiological underpinnings of PD and suggest a promising potential that warrants further exploration. Despite the lack of these comparative studies, the continuous improvement and ongoing development of molecular neuroimaging pave the road to genuinely individualized therapies in PwND and PwPD.

## Data Availability

Not applicable.

## Supplementary Materials

The following supporting information can be downloaded at: https://www.mdpi.com/article/10.3390/antiox12071411/s1, Table S1: Preclinical/experimental neuroimaging studies in PD animal models to map neuroinflammation; Table S2: PET-based neuroimaging studies in PwPD to map neuroinflammation; Table S3: MRI-based neuroimaging studies in PwPD to map neuroinflammation.

## Abbreviations

^1^H-MRSI: proton magnetic resonance spectroscopy imaging. 6-OHDA: 6-hydroxydopamine. ^17^O-MRSI: ^17^oxygen magnetic resonance spectroscopy imaging. ^31^P-MRSI: ^31^phosphorus magnetic resonance spectroscopy imaging. ASL: arterial spin labeling. aSyn: alpha-synuclein. ATP: adenosine triphosphate. BBB: blood–brain barrier. BOLD: blood-oxygen level-dependent Imaging. CB1-R/CB2-R: cannabinoid receptor type 1 or 2. CNS: central nervous system. COX: cyclooxygenase. CSF: cerebrospinal fluid. DA: dopamine. DAergic: dopaminergic. DDE: double diffusion encoding. DKI: diffusion kurtosis imaging. DTBZ: [^11^C]-dihydro tetrabenazine. DTI: diffusion tensor imaging. DCE-MRI: dynamic contrast-enhanced MRI. FA: fractional anisotropy. FAt: corrected fractional anisotropy. FWI: free-water imaging. Gd: gadolinium. GM: gray matter. GSK-3: glycogen synthase kinase 3. HCs: healthy controls. I2BS: imidazoline-2 binding sites. MAO-B: monoamine oxidase type B. MDt: corrected mean diffusivity. MERTK: proto-oncogene tyrosine-protein kinase MER. MRI: magnetic resonance imaging. MRSI: magnetic resonance spectroscopy imaging. NADH: nicotinamide adenine dinucleotide. NODDI: neurite orientation dispersion and density imaging. ODI: orientation dispersion index. OXPHOS: oxidative phosphorylation. P-gp: P-glycoprotein. P2XR7: P2X purinoreceptor 7. PCr: phosphocreatine. PD: Parkinson’s disease. PET: positron emission tomography. pSN: posterior portion of the SN. PwAD: patients with Alzheimer’s disease. PwMSAc: patients with multiple systems atrophy, cerebellar type. PwND: patients with neurodegenerative disorders. PwPD: patients with Parkinson’s disease. PwPSP: patients with progressive supranuclear palsy. QUEST-MRI: QUEnch-assiSTed MRI. RDt: corrected radial diffusivity. ROI: region-of-interest. SN: *Substantia nigra*. SPIO: superparamagnetic particles of iron oxide. TREM1: triggering receptor expressed on myeloid cells-1. TSPO: mitochondrial translocator protein. USPIO: ultrasmall superparamagnetic particles of iron oxide. WM: white matter.
