# Supplementary material for: Neuroinflammation and Mitochondrial Dysfunction in Parkinson’s Disease: Connecting Neuroimaging with Pathophysiology
Source: Antioxidants (Basel). 2023 Jul 12;12(7):1411. doi: 10.3390/antiox12071411 (PMC10375976; doi:10.3390/antiox12071411)
Supplement: Supplementary file 1 [file antioxidants-12-01411-s001.zip › antioxidants-2479834-supplementary/antioxidants-2479834_SuppT1.pdf]

Table S1. Preclinical/experimental neuroimaging studies in PD animal models to map neuroinflammation.

Authors	Study Type	Subjects	Scanner	Method	ROIs	Results/Conclusion
PET-based neuroimaging studies - Cannabinoid (CB1 receptor)						
Casteels et al., 2010	preclinical/ experimental	10 treated (quinolinic acid) rats vs. 10 non-treated rats	Concord	[^{18}F]-MK-9470	whole-brain (voxel-wise)	changes in endocannabinoid transmission, specifically for CB1 receptors in the quinolinic acid-treated rat model of PD, with mainly involvement of the caudate/putamen, but also distant regions of the motor circuitry, including the cerebellum and somatosensory cortex.
PET-based neuroimaging studies - Adenosine (A2A receptor)						
Schröder et al., 2020	preclinical/ experimental	12 healthy mice	Mediso nanoScan	[^{18}F]-FESCH	striatum	no significant difference in the striatal A2A receptor density between rotenone-treated mice and controls
MRI-based neuroimaging studies - Diffusion Kurtosis Imaging (DKI)						
Arab et al., 2019	preclinical/ experimental	20 toxin- vs. 11 saline-injected mice	Bruker 9.4 T	two-shot SE-EPI	SN, striatum, sensorimotor cortex, hippocampus, thalamus	decrease in kurtosis in SN, striatum and sensorimotor cortex
Khairnar et al., 2016	preclinical/ experimental	9 transgenic vs. 12 wt mice	Bruker 9.4 T	two-shot SE-EPI	SN, striatum, sensorimotor cortex, hippocampus, thalamus	increased kurtosis and decreased diffusivity values in GM regions such as the thalamus and sensorimotor cortex; alpha-synuclein levels positively correlated with kurtosis and negatively correlated with diffusivity
MRI-based neuroimaging studies - Dynamic Contrast-Enhanced (DCE) MRI - Gd-based						
Olmedo-Diáz et al., 2017	preclinical/ experimental	27 treated vs. 27 untreated rats	Bruker 9.4 T	T1-weighted + Gd	SN	colocalization between activated microglia, BBB leakage and iron following to 6-OHDA injection
Virel et al., 2015	preclinical/ experimental	19 treated vs. 27 untreated rats	Bruker 9.4 T	T1-weighted + Gd	whole-brain	microglia translocation without any increase in BBB permeability in PwPD
MRI-based neuroimaging studies - Dynamic Contrast-Enhanced (DCE) MRI - SPIO-based						
Virel et al., 2015	preclinical/ experimental	19 treated vs. 27 untreated rats	Bruker 9.4 T	T2*-weighted + SPIO	whole-brain	microglial translocation without BBB permeability increase

In this table, we highlight how different mentioned PET- and MRI-based neuroimaging methods have been previously used in preclinical models of PD. 6-OHDA: 6-hydroxydopamine. BBB: blood-brain-barrier. DCE: dynamic contrast-enhanced imaging. DKI: diffusion kurtosis imaging. EPI: echo-planar imaging. Gd: gadolinium. GM: grey matter. MRI: magnetic resonance imaging. PD: Parkinson’s disease. PET: positron emission tomography. SE: single-echo. SN: substantia nigra. SPIO: superparamagnetic iron oxide particles.
