# Supplementary material for: Neuroinflammation and Mitochondrial Dysfunction in Parkinson’s Disease: Connecting Neuroimaging with Pathophysiology
Source: Antioxidants (Basel). 2023 Jul 12;12(7):1411. doi: 10.3390/antiox12071411 (PMC10375976; doi:10.3390/antiox12071411)
Supplement: Supplementary file 1 [file antioxidants-12-01411-s001.zip › antioxidants-2479834-supplementary/antioxidants-2479834_SuppT2.pdf]

Table S2. PET-based neuroimaging studies in PwPD to map neuroinflammation.

Authors	Study Type	Subjects	Scanner	Radioligand	ROIs	Results/Conclusion
Astroglia cells (imidazoline-2- and MAO-B receptors)						
Wilson et al., 2019	cross-sectional	8 esPwPD vs.<br>14 lsPwPD vs.<br>14 HCs	Siemens	[^{18}F]-BU99008<br>(imdiazoliine-2)	whole-brain (ROI-wise)	evidence for the *in-vivo* role of astroglia in the initiation and progression of PD
Schönecker et al., 2019	cross-sectional	9 PwPD vs.<br>6 PwMSAp vs.<br>6 PwMSAc vs.<br>13 PwPSP	GE	[^{18}F]-THK5351 (MAO-B)	whole-brain (ROI- and voxel-wise)	combined MAO-B and tau binding of [^{18}F]-THK5351 facilitates differential diagnosis of PwAP
Cannabinoid (CB1 receptor)						
Ajalin et al., 2022	cross-sectional	16 PwPD vs.<br>10 HCs		[^{18}F]-FMPEP-d2	putamen, SN, globus pallidus, thalamus, amygdala, hippocampus, insula, corpus callosum, cerebellum, and frontal, parietal, temporal, and occipital lobes	PwPD have lower CB1 receptor availability compared with HCs, dopaminergic medication increases CB1 receptor availability in PwPD toward normal levels
Ceccarini et al., 2019	cross-sectional	38 PwPD vs.<br>10 HC	Siemens	[^{18}F]-MK-9470	whole-brain (voxel-wise)	decreased CB1 receptor availability in the prefrontal and midcingulate cortex in PwPD is strongly correlated with disturbances in executive functioning, episodic memory and visuospatial functioning
Adenosine (A2A receptor)						
Ishibashi et al., 2018	cross-sectional	10 PwPD vs.<br>6 HC	GE	[^{11}C]- preladenant	ventral striatum, caudate, and putamen	maximal A2 receptor occupancy in the ventral striatum, in the caudate, and in the putamen, no significant differences in binding profiles between PwPD and HCs
Mishina et al., 2017	cross-sectional	16 PwPD vs.<br>6 HC	SET-2400W	[^{11}C]-TMSX	putamen, caudate. frontal, temporal, and occipital lobe	distribution volume ratio of A2A receptors in the putamen is larger in PwPD w/dyskinesia compared to HC . In the drug-naïve PwPD the A2A receptors in the putamen was lower on the more affected side. A2A receptors were significantly increased after initiation of dopaminergic therapy
Ramlackhansingh et al., 2011	cross-sectional	12 PwPD	Siemens	[^{11}C]-SCH442416	caudate, putamen, thalamus	A2A receptor binding in the caudate and putamen of PwPD w/ dopaminergic therapy was higher than in PwPD w/o dopaminergic therapy
Waggan et al., 2023	cross-sectional	18 PwPD vs.<br>6 HC	ECAT HRRT	[^{11}C]-TMSX	caudate, putamen, thalamus	A2A receptors increased bilaterally in the pallidum of moderate-stage PwPD compared to HCs, increased mean striatal binding positively correlated with motor symptom severity.
Ishibashi et al., 2022	RCT	5 PwPD<br>(3 treated w/ 130 mg and<br>2 w/ 259 mg of caffeine)	GE	[^{11}C]-preladenant	striatum	mean occupancy rates of striatal A2A receptors R increased with caffeine dosage
Blood-Brain-Barrier (BBB)						
Bartels et al., 2008	cross-sectional	10 esPwPD vs.<br>10 lsPwPD vs.<br>5 PwPSP vs.<br>4 PwMSA vs.<br>9 HCs	Siemens	[^{11}C]-verapamil	whole-brain (voxel-wise)	lsPwPD and PwPSP revealed increased tracer uptake in frontal white matter regions compared to HCs. esPwPD showed lower uptake in the midbrain and frontal brain regions
Fujita et al., 2021	cross-sectional	19 PwPD vs.<br>12 HCs	GE	[^{82}]-Rubidium	bilateral anterior cingulate cortex, posterior putamen, and thalamus, right superior temporal gyrus and inferior parietal lobule	no changes in [^{82}]-Rubidium uptake between PwPD and HCs

In this table, we highlight how different mentioned PET-based neuroimaging methods have been previously used in PwPD. BBB: blood-brain-barrier. BG: basal ganglia. esPwPD: early-stage patients with Parkinson's disease. HCs: healthy controls. lsPwPD: late-stage patients with Parkinson's disease. PwAP: patients with atypical parkinsonism. PwMSAp: patients with multiple systems atrophy with predominant parkinsonism. PwMSAp: patients with multiple systems atrophy, cerebellar type. PwPD: patients with Parkinson's disease. PwPSP: patients with progressive supranuclear palsy. ROI: region of interest.
