# Supplementary material for: Neuroinflammation and Mitochondrial Dysfunction in Parkinson’s Disease: Connecting Neuroimaging with Pathophysiology
Source: Antioxidants (Basel). 2023 Jul 12;12(7):1411. doi: 10.3390/antiox12071411 (PMC10375976; doi:10.3390/antiox12071411)
Supplement: Supplementary file 1 [file antioxidants-12-01411-s001.zip › antioxidants-2479834-supplementary/antioxidants-2479834_SuppT3.pdf]

Table S3. MRI-based neuroimaging studies in PwPD to map neuroinflammation.

Author	Study Type	Subjects	Scanner	Sequence	ROIs	Outcomes	Results/Conclusion
Diffusion Kurtosis Imaging (DKI)							
Bai et al., 2021	cross-sectional	64 esPwPD vs. 44 lsPwPD vs. 64 HCs	GE 3.0 T	two-shell diffusion SP-EPI	BG	diffusion metrics	PwPD revealed progressive microstructural alterations in the subcortical nuclei detectable with DKI
Guan et al., 2019	cross-sectional	26 PwPD vs. 15 HCs	GE 3.0 T	SE-EPI	SN, RN	diffusion metrics	decreased mean kurtosis values in the SN are a surrogate marker of disease severity and may facilitate early diagnosis
Rong et al., 2022	cross-sectional	20 esPwPD vs. 15 lsPwPD vs. 20 HCs	GE 3.0 T	two-shot SE-EPI	BG, thalamus, pons, midbrain	diffusion metrics	alterations of advanced diffusion metrics in the SN may have potential in detecting esPwPD
Surova et al., 2016	cross-sectional	105 PwPD vs. 44 HC	Siemens 3.0 T	single-shot SE-EPI	BG, thalamus, pons, midbrain	diffusion metrics	PwPD patients exhibit microstructural changes in the putamen, the thalamus, and other brain regions, which are predictive for disease severity
Surova et al., 2018	longitudinal	76 PwPD vs. 38 HCs	Siemens 3.0 T	single-shot SE-EPI	BG, thalamus	diffusion metrics	microstructural changes in the putamen of PwPD can be detected over a 2-year period with DKI
Double Diffusion Encoding (DDE) Imaging							
Kamiya et al., 2020	cross-sectional	27 PwPD vs. 23 HCs	Siemens 3.0 T	LTE and PTE pairs of diffusion sensitizing gradients blocks	anterior corona radiata, internal capsule, corpus callosum, subcortical WM	diffusion metrics, within-subject variation coefficient	reductions of kurtosis in normal aging (HCs) and PwPD are likely driven by the reduction in microscopic anisotropy, microscopic anisotropy correlated with motor symptom severity in PwPD
Neurite Orientation Dispersion and Density Imaging (NODDI)							
Andica et al., 2018	cross-sectional	29 PwPD vs. 29 HCs	Siemens 3.0 T	SE-EPI, Multishell DWI	whole-brain (TBSS)	diffusion metrics	reduction in advanced diffusion metrics in the nigrostriatal pathway was more pronounced in PwPD
Andica et al., 2020	cross-sectional	29 PwPD vs. 25 HCs	Siemens 3.0 T	SE-EPI, Multishell DWI	whole-brain (TBSS)	diffusion metrics	widespread reduction in advanced diffusion metrics in PwPD
Kamagata et al., 2016	cross-sectional	58 PwPD vs. 36 HCs	Philips 3.0 T	SE-EPI	BG, SN	diffusion metrics	reduction in advanced diffusion metrics reflect lower SN neurite density
Loeherer et al., 2022	cross-sectional	33 PwPD vs. 32 HCs	Siemens 3.0 T	DWI - DTI - NODDI/DTI	whole-brain (WM tracts)	diffusion metrics	Increased advanced diffusion metrics in subcortical, cortical, and cerebellar structures in PwPD
Mitchell et al., 2019*	cross-sectional	44 PwPD vs. 21 PwMSAp vs. 26 PwPSP vs. 24 HCs	Siemens 3.0 T	Multi-shell DWI	BG, corpus callosum, thalamus, midbrain, cerebellum	diffusion metrics	NODDI-derived advanced diffusion metrics and FWI offer similar discriminability between PwPD and PwAP
Ogawa et al., 2021	cross-sectional	51 PwPD vs 23 HCs	Siemens 3.0 T	SE-EPI, Multishell DWI	whole-brain (WM tracts)	diffusion metrics	reduction in advanced diffusion metrics in the temporal WM fibers are associated with dyskinesias
Free Water Imaging (FWI)							
Andica et al., 2019	cross-sectional	20 PwPD vs. 20 HCs	Siemens 3.0 T	SE-EPI, Multishell DWI	whole-brain (TBSS)	FW, diffusion metrics	PwPD revealed lower FWI indices in anterior WM, while showing an increase in posterior WM areas; potential role of FWI as an in-vivo biomarker of disease progression and neuroinflammation
Arribarat et al., 2019	cross-sectional	18 PwPD vs. 21 HCs	Siemens 3.0 T	SE-EPI	SN	FW, T2*, diffusion metrics	T2* and FWI differences are reflecting complementary pathophysiological pathways
Gatusso et al., 2022	cross-sectional	130 PwPD vs. 58 HCs	Siemens 3.0 T	2D single-shot SE-EPI	thalamus, dorsomedial nucleus, Meynert nucleus, hippocampus, entorhinal cortex	FW, diffusion metrics	Thalamic dorsal motor nucleus FW is a progression biomarker in esPwPD

Zhang et al., 2022	cross-sectional	65 PwPD vs. 37 PwiRBD vs. 41 HCs	Siemens 3.0 T	not provided	SN	FW, Susceptibility values, diffusion metrics	QSM and FWI have similar discriminative power in the diagnosis of esPwPD, While the combination of these two methods further increases the discriminative power
Arpin et al., 2022	RCT	90 PwPD	Siemens 3.0 T	Multishell DWI	SN	FW, diffusion metrics	FW correlated with motor symptom severity in PwPD
Dynamic Contrast-Enhanced (DCE) MRI - Gd-based							
Al-Bachari et al., 2020	cross-sectional	49 PwPD vs. 46 HCs	Philips 3.0 T	T1-weighted + Gd	whole-brain	K^{trans}	higher K^{trans} in PwPD, especially in posterior WM regions, indicates BBB disruption
Ding et al., 2021	cross-sectional	398 PwPD vs. 319 PwAP vs. 371 HCs	Siemens 3.0 T	T1-weighted + Gd	whole-brain	time to peak	impaired lymphatic drainage in PwPD. Experimental results in animal models demonstrate that lymphatic drainage dysfunctions aggravate alpha-synuclein pathology and contributes to the disease progression
Dynamic Contrast-Enhanced (DCE) MRI - SPIO-based							
Sharma et al., 2022	cross-sectional	PwPD and HCs (numbers not provided)	not provided	SWI + SPIO, MICRO (Microvascular In-vivo Contrast Revealed Origins) imaging	SN	voxel intensity (vascular enhancement)	detailed mapping of SN microvasculature in PwPD possible

In this table, we highlight how different mentioned MRI-based neuroimaging methods have been previously used in PwPD. However, most of these studies did not discuss their findings concerning neuroinflammation. *This study also investigates the role of FWI in PwPD. BBB: blood-brain-barrier. BG: basal ganglia. DCE: dynamic contrast-enhanced MRI. DDE: double diffusion encoding imaging. DKI: diffusion kurtosis imaging. DWI: diffusion-weighted imaging. DTI: diffusion tensor imaging. esPwPD: early-stage patients with Parkinson's disease. FW: free water. FWI: free water imaging. Gd: gadolinium. HCs: healthy controls. lsPwPD: late-stage patients with Parkinson's disease. LTE: linear tensor encoding. MRI: magnetic resonance imaging. NODDI: neurite orientation dispersion and density imaging. PTE: planar tensor encoding. PwAP: patients with atypical parkinsonism. PwiRBD: patients with idiopathic REM-sleep behavior disorder. PwMSAp: patients with multiple systems atrophy with predominant parkinsonism. PwPD: patients with Parkinson's disease. PwPSP: patients with progressive supranuclear palsy. QSM: quantitative susceptibility-weighted imaging. RN: red nucleus. SE-EPI: spin-echo - echo planar imaging. SN: substantia nigra. SPIO: superparamagnetic iron oxide. SWI: susceptibility-weighted imaging. TBSS: tract-based spatial statistics. WM: white matter.
